# Optimization of Duck Semen Freezing Procedure and Regulation of Oxidative Stress

**DOI:** 10.3390/ani15152309

**Published:** 2025-08-06

**Authors:** Zhicheng Wang, Haotian Gu, Chunhong Zhu, Yifei Wang, Hongxiang Liu, Weitao Song, Zhiyun Tao, Wenjuan Xu, Shuangjie Zhang, Huifang Li

**Affiliations:** Jiangsu Institute of Poultry Science, Yangzhou 225125, China; 18252755263@163.com (Z.W.); wzcjips@126.com (H.G.); zhuchunhong301@126.com (C.Z.); wangjiangxian12@126.com (Y.W.); liuhx301@126.com (H.L.); songweitao301@126.com (W.S.); taozhiyuan302@163.com (Z.T.); xuwenjuan303@163.com (W.X.); zhangshuagnjie301@163.com (S.Z.)

**Keywords:** Jinding ducks, semen freezing procedure, semen quality, oxidative stress, lipid peroxidation

## Abstract

Waterfowl semen cryopreservation technology is a key link in genetic resource conservation and artificial breeding, but poultry spermatozoa, due to their unique morphology and biochemical properties, are prone to oxidative stress during freezing, resulting in a significant decrease in vitality. In this study, we first used four different freezing procedures (P1–P4) to freeze duck semen and compared their effects on duck sperm quality. Then, the changes in antioxidant indexes in semen were monitored. The results showed that program P4 (initial 7 °C/min slow descent to −35 °C, followed by 60 °C/min rapid descent to −140 °C) was significantly better than the other programs (*p* < 0.05), and its post-freezing sperm vitality reached 71.41%, and the sperm motility was 51.73%. In the P1 and P3 groups, the sperm vitality was 65.56% and 53.41%, and the sperm motility was 46.99% and 31.76%, respectively. In terms of antioxidant indexes, compared with the fresh semen group (CK), the activities of superoxide dismutase (SOD), catalase (CAT), and glutathione peroxidase (GSH-px) in the P2 group were significantly decreased (*p* < 0.05), while the activities of SOD and CAT in the P4 group showed no significant changes (*p* > 0.05) except that the activity of GSH-px was significantly decreased (*p* < 0.05). And the CAT and GSH-px activities in the P4 group were significantly higher than those in the P2 group (*p* < 0.05). The content of malondialdehyde (MDA) in the P2 group was significantly higher than that in the fresh semen group (*p* < 0.05), and there was no significant difference between the P2 group and the P4 group (*p* > 0.05). The total antioxidant capacity (T-AOC) content of the P2 and P4 groups was significantly lower than that of the fresh semen group (*p* < 0.05). The staged cooling strategy of P4 was effective in reducing the exposure time to the hypertonic environment by balancing intracellular dehydration and ice crystal inhibition, shortening the reactive oxygen species accumulation and alleviating oxidative stress injury. On the contrary, the multi-stage slow-down strategy of P2 exacerbated mitochondrial dysfunction and the oxidative stress cascade response due to prolonged cryogenic exposure time. The present study confirmed that the freezing procedure directly affects duck sperm quality by modulating the oxidative stress pathway and provides a theoretical basis for the standardization of duck semen cryopreservation technology.

## Simple Summary

Preserving duck semen through freezing is crucial for breeding and production programs, but the process has severe effects on sperm. In this study, we first used four different freezing procedures to freeze duck semen and compared their effects on duck sperm quality. The four freezing procedures are as follows: procedure 1 (P1), 5 °C → −10 °C (5 °C/min) → −130 °C (60 °C/min); procedure 2 (P2), 5 °C → 2 °C (1 °C/min, hold for 5 min) → −3 °C (10 °C/min) → −20 °C (6 °C/min) → −90 °C (10 °C/min); procedure 3 (P3), 5 °C → −44 °C (12 °C/min) → −120 °C (40 °C/min); procedure 4 (P4), 5 °C → −35 °C (7 °C/min) → −140 °C (60 °C/min). Then, according to the results of a semen quality test, the oxidative stress indicators of the control group (fresh semen) and the two groups with the highest and lowest sperm motility and vitality were measured. Our results indicated that a freezing procedure with an initial slow drop of 7 °C/min to −35 °C followed by a rapid drop of 60 °C/min to −140 °C was most suitable for duck semen freezing with minimal effect on duck sperm quality. In addition, this study confirmed that the freezing procedure directly affects duck sperm quality by modulating the oxidative stress pathway. Taken together, future research should focus on how to further improve cryopreservation procedures to reduce oxidative stress damage in duck semen, thereby improving the sustainability of the poultry industry.

## 1. Introduction

As an important pillar of the agricultural economy, waterfowl breeding plays a key role in meeting market demand, promoting breed improvement and biodiversity conservation. With the development of breeding technology, improving the reproductive efficiency of waterfowl has become a research focus. Cryopreservation of duck semen is the most practical method for long-term preservation of its genetic resources [1,2], and it is also one of the most important steps in artificial insemination technology [3] and has been widely used in mammals [4,5], but still faces challenges in the field of poultry [6]. Avian spermatozoa are extremely sensitive to temperature changes due to their unique morphology, structure, and biochemical properties [7,8] and are prone to plasma membrane rupture, acrosome damage, and oxidative stress during the freezing process, leading to a significant decrease in vitality and fertilization ability [9]. To a certain extent, the development of poultry semen cryopreservation technology is limited, resulting in the technology still being in the experimental stage [10] and being inapplicable for commercial application and the protection of genetic information and resources [11]. Therefore, optimizing the freezing procedure of avian semen and elucidating the mechanism of freezing damage are essential to break through the technological bottleneck.

In contrast to mammals, poultry spermatozoa have an elongated morphology, minimal cytoplasmic content, and a plasma membrane rich in polyunsaturated fatty acids [12,13,14]. This structure facilitates the movement of spermatozoa in the female reproductive tract but makes them highly susceptible to physical and biochemical damage during freezing [15]. Physical damage is mainly caused by the formation of intracellular ice crystals, especially in the “ice crystal danger zone” between −15 °C and −60 °C, where the mechanical stress of ice crystals can directly lead to rupture of the plasma membrane and damage to the acrosome structure [16,17,18]. Biochemical damage is closely related to freezing-induced oxidative stress. In cold environments, dysfunction of the mitochondrial respiratory chain leads to the overproduction of reactive oxygen species such as superoxide radicals (O_2_^−^) and hydrogen peroxide [H_2_O_2_] [19]. When the concentration of these oxidation products exceeds the regulatory range of the cell’s own antioxidant defense mechanisms, a series of oxidative damages are induced, including pathological changes such as lipid peroxidation of biological membranes [20,21], oxidative breaks in genetic material, and alterations in protein structure [22,23]. In addition, this process leads to impaired sperm energy metabolism, reduced ATP synthesis, and loss of sperm motility [24,25]. It has been found that the levels of malondialdehyde (MDA), a marker of lipid peroxidation, are significantly elevated and the activity of superoxide dismutase (SOD) is significantly decreased in spermatozoa after cryopreservation treatment compared to fresh sperm samples, suggesting that oxidative stress is a central mechanism leading to sperm damage during sperm cryoinjury [26,27].

Although some studies have mitigated oxidative damage through the addition of exogenous antioxidants, such as astaxanthin [3], apigenin [28], Mito-TEMPO [29], and so on, the optimization of the freezing procedure itself is still neglected. Freezing rate, as a key parameter, needs to be balanced between ice crystal formation and osmotic pressure damage. Rapid cooling (>50 °C/min), while inhibiting intracellular ice crystals, may lead to membrane lipid phase change, while slow cooling (<10 °C/min) prolongs the exposure of cells to hyperosmotic environments and exacerbates reactive oxygen species (ROS) accumulation [23,30]. Currently, programmed freezing studies on chicken semen have demonstrated that a multi-stage warming strategy, such as an initial slow descent to avoid cold shock and a later rapid descent to reduce ice crystals, can improve sperm vitality to more than 50% [31]. However, there is a paucity of studies on duck semen, and most of the available literature follows the chicken semen freezing protocol. Therefore, in this study, four differentiated freezing procedures (P1–P4) were designed for Jinding ducks, and their effects on sperm vitality, mortality, antioxidant enzyme activity, lipid peroxidation marker content, and total antioxidant capacity were systematically compared, with the aim of investigating the mechanisms of different freezing protocols on the regulation of sperm quality through the oxidative stress pathway and to provide a theoretical basis for the standardization of semen cryopreservation technology in ducks. The purpose of this study is to investigate the regulation mechanism of sperm quality through oxidative stress in different freezing protocols and to provide a theoretical basis for standardization of duck semen cryopreservation technology.

## 2. Materials and Methods

### 2.1. Ethics Statement

This research plan has been approved by the Animal Protection and Utilization Committee of Jiangsu Poultry Science Institute (Yangzhou, China; approval no. CAC-JIPS01342). All possible efforts were made to reduce the animals’ suffering to the minimum.

### 2.2. Animals and Groups

In this study, 45 healthy 43-week-old Jinding male ducks with consistent body weight and condition provided by the National Domesticated Animal Germplasm Resource Bank (2025) were selected, and all individuals were trained by the artificial massage method for 2 weeks to ensure that qualified semen could be stably collected. During the training period, the experimental ducks were housed in a single cage in a stepped cage and fed full-value feed according to the nutritional standards for laying ducks, with free access to water (calculated on the basis of 88% dry matter: crude protein, 17%; metabolizable energy, 2550 kcal/kg). Before the experiment, all ducks were screened for semen quality (e.g., motility and vitality) to exclude the influence of individual differences to ensure semen homogeneity. Then, 45 male ducks were randomly divided into a fresh semen control group (CK) and four freezing procedure treatment groups (P1–P4). Each treatment group consisted of three replicates, with three ducks per replicate (i.e., nine ducks per treatment group in total). The same males were consistently maintained in their respective groups throughout the experiment to avoid confounding effects from individual variation. The semen samples were collected in the spring, and their breeding followed the specification for healthy poultry production (GB/T32148-2015) [32].

### 2.3. Preparation of Semen Dilutions

Beltsville Poultry Semen Extender (BPSE) diluent was used, which was prepared as follows: accurately weighed D-fructose 0.5 g, monosodium glutamate 0.867 g, magnesium chloride hexahydrate 0.034 g, sodium acetate 0.43 g, sodium citrate 0.064 g, potassium dihydrogen phosphate 0.065 g, disodium hydrogen phosphate 0.4732 g, N-trimethyl-2-aminoethanesulfonic acid 0.195 g, and gradient dissolution using ddH_2_O. After the solutes were completely dissociated, the mixture was volume-determined to a final 100 mL, the pH of the solution was adjusted to 7.2–7.4, and the solution was stored at 4 °C for spare use.

### 2.4. Semen Collection and Pretreatment

The semen collection interval was 48 h, and three repetitions were performed. The time of semen collection was fixed at 9:00 a.m. daily, and semen samples were obtained by a combination of dorsal–abdominal massage and cloacal stimulation. Qualified samples had to meet the following conditions: milky white color and homogeneous texture, without fecal or urine contamination. After collection, semen samples were quickly transferred to a 15 mL sterile centrifuge tube, gently mixed with an equal volume of BPSE diluent pre-cooled at 4 °C, and equilibrated at 4 °C for 1 h without being exposed to any heat shocks and sunlight. To minimize thermal shock, we pre-warmed the 4 °C extender to match the temperature of fresh semen before mixing. Then, the diluted semen was mixed with an equal volume of 8% dimethylacetamide (DMA) (92% dilution + 8% DMA) and equilibrated at 4 °C for 30 min.

### 2.5. Semen Freezing and Thawing

The equilibrated semen was dispensed into 0.25 mL wheat tubes, and four freezing procedures were performed using a programmed cryostat (Thermo Fisher Scientific, Waltham, MA, USA, item number TSCM17PV) (Table 1). Upon completion of the program, the wheat tubes were quickly transferred to liquid nitrogen and stored for 1 h. The wheat tubes were thawed using a 37 °C water bath for 30 s, immediately followed by sperm vitality testing.

### 2.6. Semen Quality Testing

The detection method was in accordance with the methods of testing of poultry semen quality (NY/T 4047-2021) [33]. Sperm vitality and motility were detected using a fully automated sperm analyzer (Nanjing Songjing Tianlun Biotechnology Co., Ltd. (Nanjing, China), item number MDO300A). Sperm motility (%) = [(Number of progressively motile sperm + Number of non-progressively motile sperm)/Total number of sperm counted] × 100. The sperm vitality test was performed as follows: A 10 μL aliquot of eosin–nigrosin stain was placed on the right side of a glass slide, mixed thoroughly with 10 μL of the sample, and allowed to stain for approximately 1 min. Another glass slide (spreader slide) was held by its long edge with the right hand, and its short edge was gently touched to the stained sample at a 45° angle. The spreader slide was then smoothly drawn to the left in one swift motion (within 1 s) to create a thin, uniform smear. The smear was air-dried at room temperature. Approximately 200 spermatozoa from different fields were counted to determine the number of live (unstained) and dead (stained) sperm. Sperm vitality (%) = [Live sperm count/(Live sperm count + Dead sperm count)] × 100.

### 2.7. Measurement of Oxidative Stress Indicators

According to the results of the semen quality test, the oxidative stress indicators of the control group and the two groups with the highest and lowest sperm motility and vitality were measured. Semen samples were taken, and the following indicators were measured: total SOD, CAT, GSH-Px activity, and MDA and T-AOC content, which were detected by using the kit of Nanjing Jiancheng Bioengineering Research Institute Co., Ltd. (Nanjing, China), and the results were standardized to protein concentration.

### 2.8. Data Processing and Analysis

The experimental data were expressed as mean ± standard deviation and were analyzed by one-way analysis of variance (ANOVA) using SPSS 20.0 software and differences between groups were tested by the Tukey HSD method with the level of significance set at *p* < 0.05. Graphs were drawn using GraphPad Prism 9.0.

## 3. Results

### 3.1. Sperm Vitality

**Figure 1:**
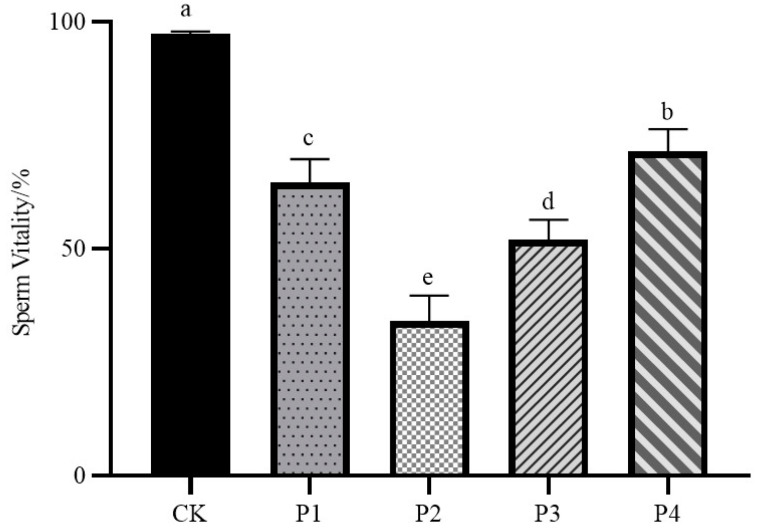
Impact of different freezing procedures on sperm vitality. Abbreviations: CK, fresh semen group; P1, freezing procedures 1 group; P2, freezing procedures 2 group; P3, freezing procedures 3 group; P4, freezing procedures 4 group. Significant differences are indicated by different lowercase letters (*p* < 0.05).

As shown in Figure 1, the sperm vitality of the fresh semen control group was 97.4%, which was significantly different from that of all four freezing procedure treatment groups (*p* < 0.05); the sperm vitality of the P4 group was the highest among the four freezing procedure treatment groups, which was 71.41%; the sperm vitality of the P2 group was the lowest among the four freezing procedure treatment groups, which was 34.28%; and the mean sperm vitality of the P1 group and the P3 group were in the middle of the range, which were 65.56% and 53.41%, respectively.

### 3.2. Sperm Motility

**Figure 2:**
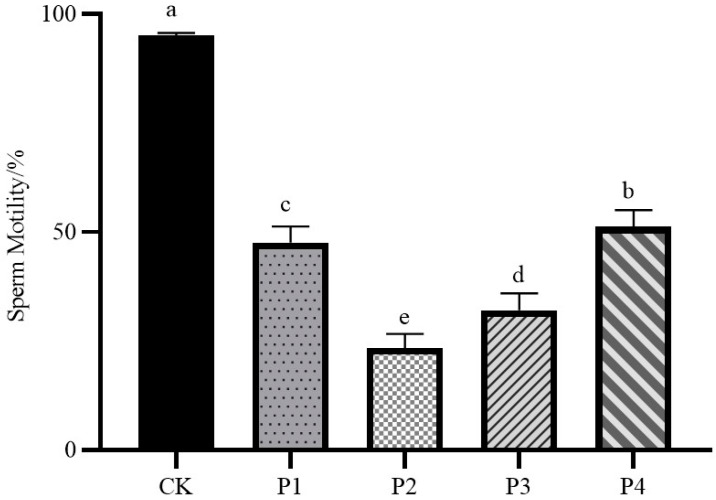
Impact of different freezing procedures on sperm motility. Abbreviations: CK, fresh semen group; P1, freezing procedures 1 group; P2, freezing procedures 2 group; P3, freezing procedures 3 group; P4, freezing procedures 4 group. Significant differences are indicated by different lowercase letters (*p* < 0.05).

As shown in Figure 2, the sperm motility of the fresh semen control group was 95.2%, which was significantly different from that of the four freezing procedure treatment groups (*p* < 0.05); the sperm motility of the P4 group was the highest among the four freezing procedure treatment groups, 51.73%; the sperm motility of the P2 group was the lowest among the four freezing procedure treatment groups, 22.69%; and the sperm motility of the P1 group and the P3 group was in the middle of the average, 46.99% and 31.76%.

### 3.3. Superoxide Dismutase (SOD)

**Figure 3:**
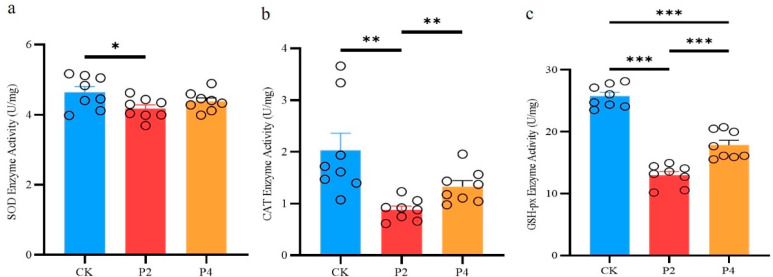
Impact of different freezing procedures on the activity of sperm antioxidant enzymes ((**a**), SOD enzyme activity; (**b**), CAT enzyme activity; (**c**), GSH-px enzyme activity). Abbreviations: CK, fresh semen group; P2, freezing procedures 2 group; P4, freezing procedures 4 group. The * symbol denotes a significant difference (*p* < 0.05). The ** symbol denotes (*p* < 0.01). The *** symbol denotes (*p* < 0.001).

As shown in Figure 3a, compared with the control group, the SOD activity was significantly reduced in the P2 group (*p* < 0.05), while there was no significant difference in the P4 group (*p* > 0.05).

### 3.4. Catalase (CAT)

As shown in Figure 3b, CAT activity was significantly decreased in the P2 group (*p* < 0.05) and not significantly altered in the P4 group when compared to the control group (*p* > 0.05); but CAT activity was significantly increased in the P4 group when compared to the P2 group (*p* < 0.05).

### 3.5. Glutathione Peroxidase (GSH-Px)

The results showed that GSH-Px activity was significantly decreased in both the P2 and P4 groups compared to the control group, but GSH-Px activity in the P4 group was significantly higher than that in the P2 group (*p* < 0.05) (Figure 3c).

### 3.6. Malondialdehyde (MDA) and Total Antioxidant Capacity (T-AOC)

**Figure 4:**
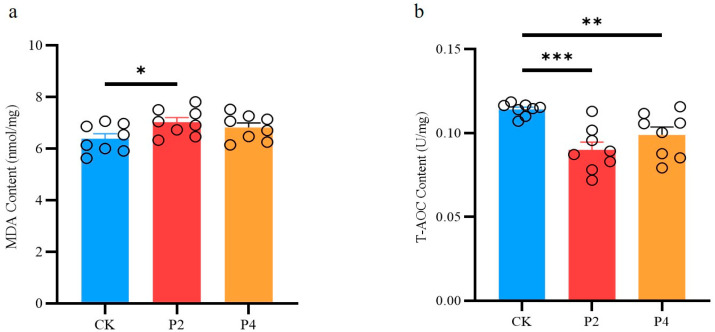
Effects of different freezing methods on MDA and T-AOC contents in semen ((**a**), MDA content; (**b**), TAOC content). Abbreviations: CK, fresh semen group; P2, freezing procedures 2 group; P4, freezing procedures 4 group. The * symbol denotes a significant difference (*p* < 0.05). The ** symbol denotes (*p* < 0.01). The *** symbol denotes (*p* < 0.001).

The results of this study showed that compared with the control group, the MDA level in the P2 group significantly increased (*p* < 0.05), while there was no significant change in the P4 group (*p* > 0.05) (Figure 4a); the T-AOC content in both the P2 and P4 groups significantly decreased (*p* < 0.05), but the decreasing trend in the P4 group was lower than that in the P2 group (Figure 4b).

## 4. Discussion

In this study, we systematically compared the effects of four differentiated freezing procedures on sperm vitality, motility, and oxidative stress indexes in Jinding ducks, revealing the critical roles of freezing rate and cooling strategy on sperm quality by modulating the oxidative stress pathway. The results confirmed that freezing program P4 performed optimally in maintaining sperm vitality and motility, while freezing program P2 led to a significant decrease in sperm vitality and motility. Physical and biochemical damage to spermatozoa during cryopreservation is a central factor in the reduction in their vitality [15,34]. Procedure P4 used a two-stage cooling strategy, with an initial slow descent to −35 °C at 7 °C/min followed by a rapid descent to −140 °C at 60 °C/min. This protocol achieved an optimized effect by balancing ice crystal generation with osmotic pressure damage. In the initial slow descent phase, the cells were gradually dehydrated to reduce the internal free water content, which in turn effectively inhibited ice crystal production; the subsequent rapid descent phase shortened the spermatozoa’s exposure to the hyperosmotic environment and reduced the risk of ROS accumulation, which is consistent with the optimization of the multi-stage variable temperature strategy in chicken and drake semen freezing studies [7,11]. Procedure P2 used a multi-stage slow descent, which, although aimed at avoiding cold shock, resulted in increased mitochondrial dysfunction due to prolonged exposure. Mitochondria, as the main source of ROS, undergo leakage of the electron transport chain by the absence of their membrane potential, causing the accumulation of superoxide anion (O_2_^−^) [35,36,37]. The results of this study showed that the P2 group had significantly higher MDA content and significantly lower activities of the antioxidant enzymes SOD, CAT, and GSH-Px, suggesting that oxidative stress was the main driver of their sperm damage. In contrast, the MDA content of the P4 group did not change significantly, and the CAT activity was significantly higher than that of the P2 group, suggesting that its cooling strategy effectively alleviated the lipid peroxidation reaction.

The synergistic effect of the cryoprotectant DMA should not be overlooked. DMA, as a permeable protectant, reduces cryoinjury by lowering the freezing point and stabilizing the membrane structure [38,39]. However, its protective effect is highly dependent on matching the cooling rate. The rapid terminal cooling of program P4 may reduce the interference of DMA with membrane lipids by shortening its contact time with spermatozoa and reducing its potential toxicity, whereas the slow-cooling process of program P2 may lead to over-permeation of DMA within the cell, triggering an osmotic pressure imbalance [40]. In addition, sperm vitality in the P2 group was significantly lower than that in the chicken study, despite the use of a similar retardation protocol, which may be due to the fact that the plasma membrane of duck spermatozoa is rich in polyunsaturated fatty acids [41], which are more sensitive to ROS attack.

The dynamic balance of the antioxidant system during the freezing process is crucial for sperm survival [31]. But the capacity of sperm to synthesize antioxidants is limited [42]. The antioxidant defense system basically relies on the enzymes present in seminal plasma. Among the main enzymes are glutathione peroxidase (GSH-Px), superoxide dismutase (SOD), and catalase (CAT) [4]. In this study, we found that the SOD activity in the P4 group was not significantly different from that of fresh semen, suggesting that this freezing procedure effectively inhibited O_2_^−^ production, whereas the CAT activity was significantly higher than that in the P2 group, indicating that the scavenging ability of H_2_O_2_ was preserved. This result is consistent with the physiological function of the SOD-CAT cascade reaction [43], in which SOD converts O_2_^−^ to H_2_O_2_ and oxygen [44], and CAT or GSH-Px further breaks it down into harmless water and oxygen [45]. However, GSH-Px activity remained significantly lower in the P4 group than in the control group, suggesting that the glutathione system may be difficult to fully recover during freezing. The regeneration of glutathione as an important non-enzymatic antioxidant depends on the supply of NADPH, and the mitochondrial dysfunction caused by freezing may limit the efficacy of this pathway [46], and in the future, the addition of exogenous glutathione to the dilution solution may be considered to compensate for the lack of an endogenous antioxidant system.

In summary, the present study confirmed that freezing procedures significantly affected the sperm quality of Jinding ducks by regulating the oxidative stress pathway, in which procedure P4 was outstanding in inhibiting ROS production and maintaining antioxidant enzyme activities due to its optimized cooling rate and staging strategy. This finding provides a reference for the standardization of duck semen cryopreservation technology and helps to promote the conservation of waterfowl genetic resources and the industrial application of artificial insemination technology.

## 5. Conclusions

In conclusion, the P4 protocol demonstrated the best performance, with the least negative impact on sperm motility and viability. Its staged cooling strategy effectively maintained antioxidant enzyme activity and mitigated lipid peroxidation damage. This study confirmed that P4 optimized the freezing effect by regulating the oxidative stress pathway, which provided a theoretical basis for the standardization of duck semen cryopreservation.

## Figures and Tables

**Table 1:** Parameters of freezing procedures.

Programs	Steps to Cool Down
P1	5 °C → −10 °C (5 °C/min) → −130 °C (60 °C/min)
P2	5 °C → 2 °C (1 °C/min, hold for 5 min) → −3 °C (10 °C/min) → −20 °C (6 °C/min) → −90 °C (10 °C/min)
P3	5 °C → −44 °C (12 °C/min) → −120 °C (40 °C/min)
P4	5 °C → −35 °C (7 °C/min) → −140 °C (60 °C/min)

## Data Availability

The data in this study can be obtained upon reasonable request to the corresponding author.
